# Relationship between vaginal microecology and human papillomavirus infection as well as cervical intraepithelial neoplasia in 2,147 women from Wenzhou, the southeast of China

**DOI:** 10.3389/fonc.2023.1306376

**Published:** 2024-01-03

**Authors:** Lejing Zang, Renqian Feng, Yitong Huang, Jiahe Huang, Yan Hu

**Affiliations:** ^1^ Department of Gynecological Oncology, Wenzhou Central Hospital, Wenzhou, China; ^2^ Department of Gynecology, First Affiliated Hospital of Wenzhou Medical University, Wenzhou, China

**Keywords:** vaginal microecology, human papillomavirus, cervical intraepithelial neoplasia, cervical cancer, probiotics

## Abstract

**Objective:**

The female reproductive tract is a significant microecological region, and its micro-environment can directly affect women’s cervical health. This research aimed to investigate the effect of vaginal microecology on human papillomavirus (HPV) infection and cervical intraepithelial neoplasia(CIN).

**Methods:**

A retrospective cohort study enrolling 2,147 women who underwent a colposcopic examination between August 2021 and August 2022 was conducted. The relationship between vaginal microecology and HPV infection as well as cervical lesions were assessed using the chi-square test, univariate and multivariate logistic regression analyses, and Cochran-Armitage trend test.

**Results:**

HPV infection was linked to the imbalance of vaginal microecology [odds ratio (OR)=3.00, 95% confidence interval (CI)=1.66–5.43; P<0.001]. Clue cell (OR=1.59, 95% CI=0.99–2.54; P=0.054) and sialidase (OR=1.54, 95% CI=1.01–2.35; P<0.046) were considered as significant risk factors for HPV infection. Further analysis showed that vaginal microecological disorder was more likely to be detected in patients infected with HPV 16/18 subtypes (OR=9.86, 95% CI=2.37–41.80; P=0.002). Although there was no significant correlation between the incidence of vaginal microecological disorder and the severity of cervical lesions (P > 0.05), the proportions of abnormal PH value (OR=2.6, 95% CI=1.63–10.42; P=0.001) and abnormal vaginal cleanliness (OR=2.6, 95% CI=1.36–4.0; P= 0.004) increased as the histological stage progressed.

**Conclusion:**

Vaginal microecology associates with HPV infection and the progression of cervical lesions. Detection of vaginal secretion may contribute to the development of targets for micro-environmental modulation with probiotics and the reduction of the incidence of cervical cancer.

## 1 Introduction

Cervical cancer (CC) is the second most common malignant tumor in females worldwide, after breast cancer (1). Human papillomavirus (HPV) infection, especially persistent high-risk human papillomavirus (HR-HPV) infection, plays a crucial role in the occurrence of pre-invasive precursors and even cervical cancer. According to epidemiological researches, the lifetime risk of acquiring human HPV infection exceeds 70% in sexually active women, and the infection in a considerable proportion of them regresses within 2 years after spontaneous clearing by innate immune responses (2). Long-term retention of HPV may not be easily eliminated due to various factors such as the menopausal status, immune deficiency, multiple sexual partners, vaginal microecological abnormality, etc., leading to cervical dysplasia (3–5). Early detection and timely treatment of sustained HPV infection and cervical lesions timely may be one of the principal means to prevent further carcinogenesis.

In recent years, increasing attention has been paid to identifying risk factors for HPV infection and CIN. The vagina and cervix are the first lines of physical and immunological defense against foreign microorganisms, such as viruses and bacteria. Vaginal microecology is relevant to its anatomical structure, local vaginal flora and its metabolites, immunological factors, and even endocrine condition. Under normal circumstances, the microecological flora in the vagina is dominated by lactobacillus, which can produce lactic acid, bacteriocins and reactive oxygen species (ROS) (5). Once the vaginal microbiota disorders and the local immunity gets weakened, exogenous microorganisms invade the female reproductive tract, causing infectious and inflammatory processes and increasing the risk of genital tract diseases and even cancer. Clinically, we usually evaluate the vaginal microecology by testing the morphology and function of vaginal secretion, including pathogens (trichomonas, fungus and clue cell), vagina cleanness, vaginal PH, H_2_O_2_, leukocyte esterase and sialidase respectively. It has been proved previously that HPV infection and CIN may relate to a changed flora structure and environmental dysregulation (3). Aiming at exploring the relationship between vaginal microecology and HPV infection as well as CIN, this study provides a basis for regulating the vaginal microecological environment, preventing HPV infection, and hampering the development of cervical lesions.

## 2 Materials and methods

### 2.1 Study population

This retrospective study included 2,147 women who were transferred for the colposcopic examination as per ASCCP guidelines at the First Affiliated Hospital of Wenzhou Medical University between August 2021 and August 2022 (6). The inclusion criteria and exclusion criteria in this study were as follows. Inclusion criteria: (1) Women with a history of sexual life. (2) Women underwent a colposcopic examination. (3) No sexual activity within 3 days before sampling. (4) No vaginal irrigation or administration within 7 days before sampling. Exclusion criteria: (1) Pregnant or in the lactating period. (2) Women with a history of HPV preventive vaccination. (3) Women received treatment of HPV infection or cervical lesions in the past. The designing and reporting of this study followed STROBE guidelines. This study was approved by the Ethics Committee of the First Affiliated Hospital of Wenzhou Medical University, and all participants obtained informed consent.

### 2.2 Vaginal microecology testing

All participants were advised to restrain from sex for at least 3 days and required not to use vaginal medication or take vaginal treatment for 7 days before the examination. The secretion was collected from the vaginal wall by using a gynecological brush (Dirui Medical Technology Co., Ltd). The standardized specimen was observed under a manual microscope and tested by using a vaginal secretions analysis strip (Dirui Medical Technology Co., Ltd). According to the National Clinical Laboratory Operating Guidelines, pathogens(trichomonas, fungus and clue cell)-positive results, vaginal cleanness grade III~IV, vaginal PH>4.5, H_2_O_2_>2 μmol, sialidase>7 u/mL and leucocyte esterase>7 u/mL were indicators of abnormal results. As for repeated sampling, the worst detection result before medication was considered the final outcome. Diagnosis of vaginal microecology disorder was made when any item mentioned above was abnormal.

### 2.3 HPV genotyping

Specimens for HPV genotyping were gathered from the endocervix by using a disposable sterile cervical brush and inoculated into a nucleic acid genotyping kit (Jiangsu Jianyou Medical Technology Co., Ltd.). The sample was subject to laboratory measurement including flow cytometry testing and fluorescence *in-situ* hybridization. Generally, there are 18 HR-HPV genotypes(i.e., HPV16, 18, 26, 31, 33, 35, 39, 45, 51, 52, 53, 56, 58, 59, 66, 68, 73, and 82) and 8 low-risk HPV (LR-HPV) genotypes (i.e., HPV6, 11, 40, 42, 44, 55, 61, and 83).

### 2.4 Cervical cytology

Specimens for cervical cytology were also collected from the endocervix by using disposable cervical sampler and stored in PreservCyt solution (Ningbo HLS Medical Products Co., Ltd.). Pathological slides were automatically made by a ThinPrep 2000 system (Beijing Hologic Technology Co. Ltd) and then reviewed by two experienced pathologists. The cytological diagnosis was established based on Bethesda criteria 2001. Specifically, the diagnostic results were classified into negative for no intra-epithelial lesion cells and malignancy (NILM), atypical squamous cells of undetermined significance (ASCUS), atypical squamous cells, exclude high-grade squamous epithelial lesion (ASC-H), low-grade squamous intraepithelial lesion (LSIL), high-grade squamous intraepithelial lesion (HSIL), atypical glandular cells (AGC), and carcinoma (CC), which included squamous cell carcinoma (SCC) and adenocarcinoma (AC) (7).

### 2.5 Colposcopy and histology

Indications and standards for colposcopy from the ASCCP guidelines were adopted in this study (8). The biopsy was performed after specimens were stained with pigment, and endocervical canal curettage (ECC) was an alternative if necessary. According to the 2014 WHO classification for cervical precancerous lesions, cervical tissues were pathologically divided into the following categories: with normal limits (WNL), LSIL, HSIL (CIN II/CIN III), and cervical cancer (9). Two independent histological experts in cervical histopathology were involved in the classification of the lesions. The highest grade of the cervical lesion was regarded as the final diagnosis if the results were different in the multipoint biopsy.

### 2.6 Statistical analysis

All statistical computations were performed using SPSS Version 20.0 (IBM Corp., Armonk, NY, USA). Continuous variables were presented with mean and standard deviation (SD), and categorical variables were indicated by numbers and percentages. Chi-square test, univariate and multivariate logistic regression analyses, and Cochran-Armitage trend test were performed to explore the correlation of vaginal microecology with HPV infection and CIN, being expressed with 95% confidence intervals (CI) and relative risk [odds ratio (OR)]. P-values (from two-sided tests) < 0.05 were considered to indicate statistical significance.

## 3 Results

### 3.1 Patient characteristics

**Figure 1:**
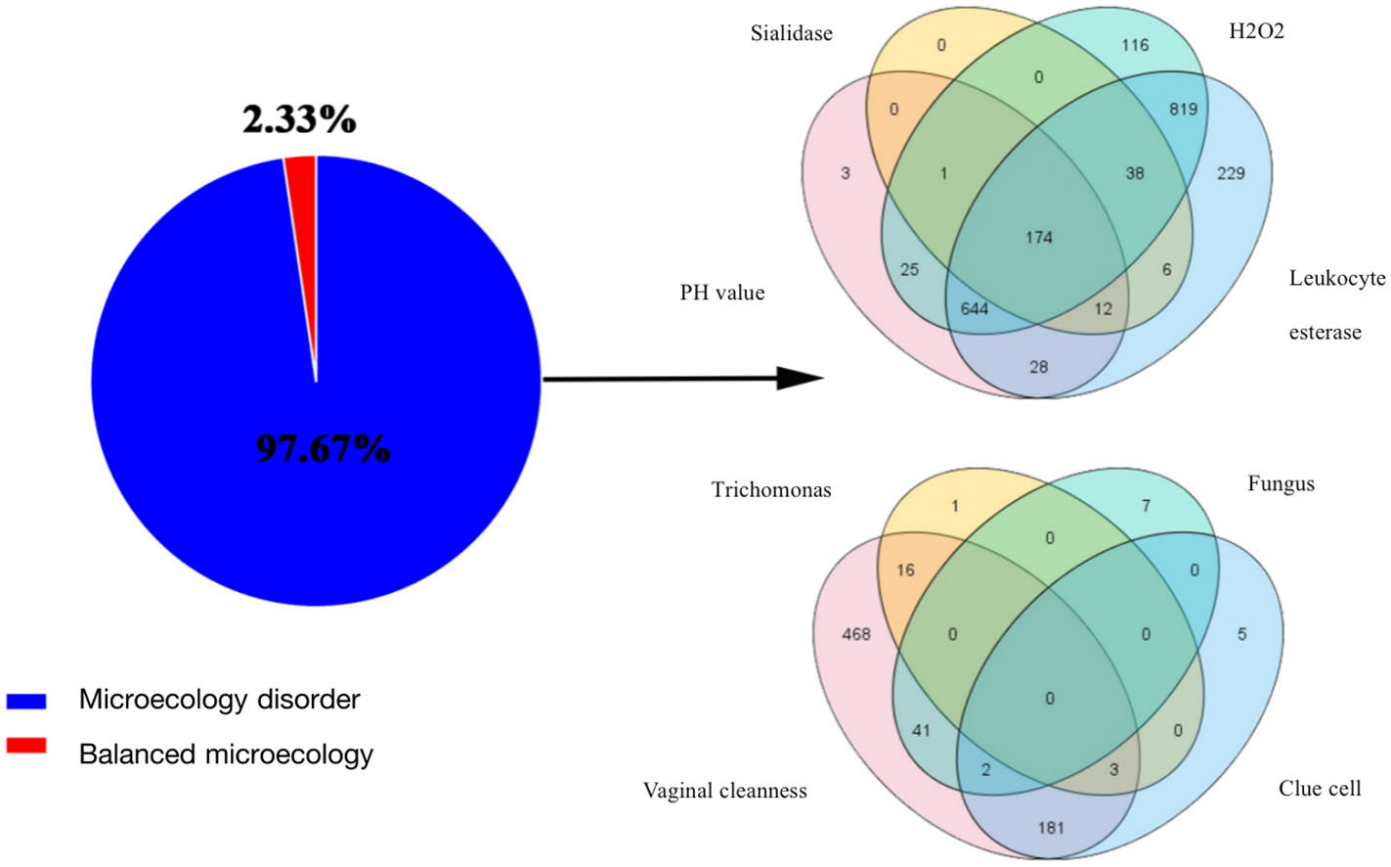
Results of vaginal microecology test. The pie chart showed the proportion of vaginal microecology disorder; The venn diagrams represented the shared and unique taxa from the perspective of function (PH, H_2_O_2_, sialidase, and leukocyte esterase) and morphology (vaginal cleanness, trichomonas, fungus and clue cell).

**Figure 2:**
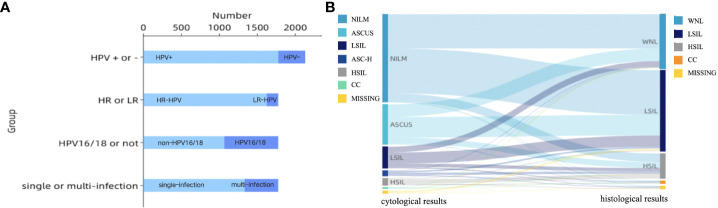
**(A)** Characteristics of HPV infection; HPV, human papillomavirus infection; HR-HPV, high-risk human papillomavirus infection; LR-HPV, low-risk human papillomavirus infection; **(B)** Sankey diagram of cervical lesions per- and post-colposcopy. The left column showed cytological results: NILM, ASCUS, LSIL, ASC-H, HSIL, CC, MISSING (n = 1134, 520, 280, 72, 92, 14, 35); The right column showed the histological results: WNL, LSIL, HSIL, CC, MISSING (n = 691, 1052, 324, 39, 41).

Clinical characteristics of patients are described in **Table 1**. Almost all patients (n = 2097, 97.7%) had vaginal microecological disorders. Among them, 887 (41.3%) women tested abnormal for vaginal PH, 1817 (84.6%) women positive for H_2_O_2_, 231(10.8%) women positive for sialidase, 1950 (90.8%) women positive for leukocyte esterase, and 711 (33.1%) women grade III~IV for vagina cleanness. Moreover, trichomonas, fungus and clue cell were detected in 20 (0.9%), 50 (2.3%) and 191 (8.9%) women, respectively (**Figure 1**). Among 1774 (82.6%) patients infected with HPV, 1622 (91.4%) had HR-HPV, 709(40.0%) had HPV 16/18, and 41 (24.9%) were infected with multiple genotypes (**Figure 2A**). Less than half of the patients (n=978, 45.6%) had cytological abnormalities, among whom ASC-US, LSIL, ASC-H, HSIL, and AGC accounted for 24.2% (n=520), 13.0% (n=280), 3.4% (n=72), 4.3% (n=92) and 0.7% (n=14), respectively. After being biopsied under colposcopy, 1415 patients (65.9%) were pathologically confirmed to have cervical lesions, and the majority of them were LSIL (n=1052, 49.0%) (**Figure 2B**).

**Table 1:** Clinical characteristics.

	group	x ± s/n, n(%)
Age		44.6 ± 10.9
PH value	≤4.5	1260 (58.7)
	>4.5	887 (41.3)
H_2_O_2_	negative	330 (15.4)
	positive	1817 (84.6)
Sialidase	negative	1916 (89.2)
	positive	231 (10.8)
Leukocyte esterase	negative	197(9.2)
	positive	1950 (90.8)
Vagina cleanness	I~II	1435 (66.8)
	III~IV	711 (33.1)
	missing	1 (0.1)
Trichomonas	no	2127 (99.1)
	yes	20 (0.9)
Fungus	no	2097 (97.7)
	yes	50 (2.3)
Clue cell	no	1956 (91.1)
	yes	191 (8.9)
HPV	negative	355 (16.5)
	positive	1774 (82.6)
	missing	18 (0.8)
HPV infection	LR-HPV	152 (8.6)
	HR-HPV	1622 (91.4)
HPV infection	HPV16/18	709 (40.0)
	Non-HPV16/18	1065 (60.0)
HPV infection	single infection	1333 (75.1)
	multiple infection	441 (24.9)
Cervical cytology	NILM	1134 (52.8)
	ASC-US	520 (24.2)
	LSIL	280 (13.0)
	ASC-H	72 (3.4)
	HSIL	92 (4.3)
	AGC	14 (0.7)
	missing	35 (1.6)
Colposcopic biopsy	WNL	691 (32.2)
	LSIL	1052 (49.0)
	HSIL	324 (15.1)
	CC	39 (1.8)
	Missing	41 (1.9)

### 3.2 Association between vaginal microecology and HPV infection

Based on the results of HPV genotyping, the cohort was divided into HPV-positive group (n=1774) and HPV-negative group (n=355). Univariate logistic regression analysis showed that 1,742 (98.3%) patients had vaginal microecology disorder in the HPV-positive group, which was observably higher than that in the HPV-negative group (94.9%) (χ²=14.5, P<0.001; OR=3.00, 95% CI=1.66-5.43). Further multivariate logistic regression analysis demonstrated that clue cell (OR=1.59, 95% CI=0.99–2.54; P=0.049) and sialidase (OR=1.54, 95% CI=1.01–2.35; P=0.046) were significant risk factors for HPV infection after confounding factors such as age and cytological results were controlled (**Tables 2**, **3**). To explore the relationships between vaginal microecology and different patterns of HPV infection, HPV-positive individuals (n=1774) were further stratified into 3 pairs of two mutually exclusive groups, namely, (1.1) infection with HR-HPV (n=1622) and (1.2) infection with LR-HPV (n =152); (2.1) infection with multi-type HPV (n=441) and (2.2) infection with single-type HPV (n= 1333); (3.1) infection with HPV 16/18 (n=709) and (3.2) infection with other HPV genotypes (n=1065). The analysis results showed that HPV 16/18 increased the incidence of vaginal microecology disorder by over nine folds (OR=9.96, 95% CI=2.37–41.80; P=0.002). Nevertheless, there were no obvious differences in vaginal microecological environment between subgroups with HR-HPV and LR-HPV patients(P=1.00), and between subgroups infected with multi-type HPV and single-type HPV(P=0.589) (**Table 4**).

**Table 2:** Association between vaginal microecology factors and HPV infection.

Factors		HPV status/n, (n%)		X^2 a^	P ^a^	OR ^a^	95%CI ^a^
		HPV positive	HPV negative				
Vaginal microecology disorder ^b^		1742 (98.3)	337 (94.9)	14.51	<0.001^*^	3.00	1.66-5.43
Morphological Evaluation	Vaginal cleanness ^c^	597 (33.7)	110 (31.0)	0.96	0.327	1.13	0.88-1.45
	Trichomonas	17 (1.0)	3 (0.8)	0.05	0.812	1.14	0.33-3.90
	Fungus	40 (2.3)	9 (2.5)	0.10	0.748	0.89	0.43-1.84
	Clue cell	168 (9.5)	23 (6.5)	3.24	0.072	1.51	0.96-2.37
Functional Evaluation	PH	741 (41.8)	140 (39.4)	0.66	0.415	1.10	0.87-1.39
	H_2_O_2_	1505 (84.8)	295 (83.1)	0.68	0.408	1.14	0.84-1.55
	Sialidase	202 (11.4)	29 (8.2)	3.17	0.075	1.45	0.96-2.17
	Leukocyte esterase	1621 (91.4)	315 (88.7)	2.51	0.113	1.35	0.93-1.95

a X^2^, P were calculated by Chi-square test; OR and 95%CI were calculated by univariate logistic regression analysis.

b The diagnosis of microecology disorder was made if any factor above was abnormal.

c Vaginal cleanness I~II were defined as normal and III~IV were defined as abnormal.

*P < 0.05.

**Table 3:** Factors associated with vaginal microecology disorder in HPV-positive woman.

	P ^a^	OR ^a^	95%CI ^a^
Clue cell	0.049 ^*^	1.59	1.66-5.43
Sialidase	0.046 ^*^	1.54	1.01–2.35

a P, OR and 95%CI were calculated by multivariate logistic regression analysis. *P < 0.05.

**Table 4:** Association between vaginal microecology factors and different patterns of HPV infection.

	Vaginal microecology disorder ^b^ (n, n%)	P ^a^	OR ^a^	95%CI ^a^
HPV positive	1742 (98.3)	<0.001 ^*^	3.00	1.16-5.43
HPV negative	337 (94.9)			
HPV16/18	707 (99.7)	0.002 ^*^	9.96	2.37-41.80
Non-HPV16/18	1035 (97.2)			
HR-HPV	1593 (98.2)	1.00	1.15	0.34-3.81
LR-HPV	149 (98.0)			
Single infection	1310 (98.3)	0.589	0.81	0.37-1.30
Multiple infection	432 (98.0)			

a P, OR, and 95%CI were calculated by univariate logistic regression analysis.

b The diagnosis of microecology disorder was made if any factor above was abnormal.

*P < 0.05.

### 3.3 Association between vaginal microecology and cervical lesions

**Figure 3:**
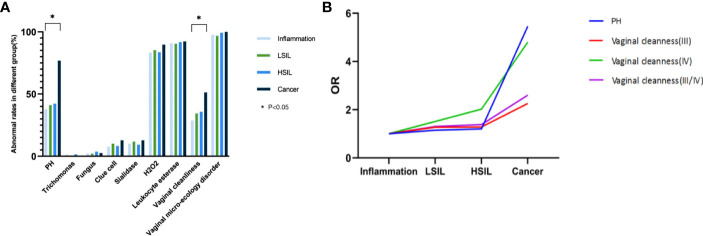
**(A)** Association between vaginal micro-ecology and cervical lesions (histology), *P < 0.05; **(B)** Trend of OR of vaginal cleanness and PH value with the progression of cervical histology, *P < 0.05.

Cochran-Armitage trend test showed that there was no significant correlation between the incidence of vaginal microecology disorder and the severity of the cervical lesion confirmed by histological pathology (P=0.279). However, the latter was roughly proportional to the proportions of patients with abnormal vaginal cleanness (Z=-3.31, P<0.001) and PH value (Z=-3.22, P=0.001) (**Table 5**, **Figure 3A**). It was pertinent to note that the abnormal vaginal cleanness and PH value were detected in all patients diagnosed with cervical cancer (n=39). Further analysis indicated that high PH value (OR=5.46, 95% CI=2.55-11.68; P<0.001) and abnormal vaginal cleanness (OR=2.60, 95% CI=1.36-4.00; P=0.004) of vaginal discharge were significantly more likely to be detected in patients with cervical cancer, especially in comparison with the general female population. Females with cervical cancer tended to have a cleanness grade of IV (**Figure 3B**). However, the chi-square test of vaginal micro-environmental factors and cytological outcomes did not identify any particular association between them (P>0.05).

**Table 5:** Association between cervical histology and vaginal microecology factors.

Factors		Cervical histology/n, (n%)				Z ^a^	P ^a^
		WNL	LSIL	HSIL	CC		
Vaginal microecology disorder ^b^		676 (97.8)	1019 (97.0)	322 (99.4)	39 (100.0)	-1.08	0.279
Morphological Evaluation	Vaginal cleanness ^c^	199 (28.8)	363 (34.5)	116 (35.8)	20 (51.3)	-3.34	<0.001 ^**^
	Trichomonas	8 (1.2)	6 (0.6)	5 (1.5)	0 (0.0)	0.12	0.902
	Fungus	15 (2.2)	21 (2.0)	12 (3.7)	1 (2.6)	-1.13	0.259
	Clue cell	53 (7.7)	105 (10.0)	27 (8.3)	5 (12.8)	-1.05	0.296
Functional Evaluation	PH	262 (37.9)	433 (41.2)	137 (42.3)	30 (76.9)	-3.22	0.001 ^*^
	H_2_O_2_	576 (83.4)	896 (85.2)	271 (83.6)	35 (89.7)	-0.74	0.461
	Sialidase	70 (10.1)	123 (11.7)	30 (9.3)	5 (12.8)	-0.12	0.902
	Leukocyte esterase	628 (90.9)	950 (90.3)	297 (91.7)	36 (92.3)	-0.33	0.74

a Z and P were calculated by Cochran-Armitage trend test.

b The diagnosis of microecology disorder was made if any factor above was abnormal.

c Vaginal cleanness I~II were defined as normal and III~IV were defined as abnormal.

*P < 0.05 **P < 0.001.

## 4 Discussion

Cervical cancer is acknowledged the most common malignant tumor in the female reproductive tract. The persistent infection with HR-HPV, especially the HPV16/18, is considered to be associated with developing cervical lesions and their recurrence after treatment (10–12). Increasing evidence suggests that local cervicovaginal factors may relate to HPV infection and following cervical lesions to a great extent. In-depth exploration of the human microecology system demonstrates that vaginal flora and its metabolites may play a vital role in maintaining the stability of the vaginal microecological environment. In this retrospective study, vaginal micro-environmental factors were found to associate with HPV infection and the development of the cervical lesions.

Bacterial vaginosis (BV), trichomonas vaginitis (TV), and vulvovaginal candidiasis (VVC) are the most common vaginal infections, which are proven related to HPV infection in previous studies (3). The research by Wang et al. (13) that enrolled 4,449 women revealed that BV and TV were closely associated with HR-HPV infection (P<0.05). Another retrospective analysis further illustrated a statistically significant difference in the proportion of BV among subgroups with HPV16/18 and non-HPV16/18 (P<0.05) (4). Nevertheless, some studies indicated that VVC did not increase the risk of HPV infection. According to their speculation, candida infection might strongly boost the immune response by promoting T cell proliferation, but the specific mechanism was still incompletely understood (14). Our results suggested that the maladjustment of vaginal microecology, especially the abnormalities in clue cell and sialidase, possibly, led to HPV infection, particularly HPV 16/18 genotypes. The detection rate of clue cell, one of the diagnostic indicators of BV, in HPV-positive patients was 0.51 times higher than that in HPV-negative patients. It validates that clue cell may serve as an indicator of viral infection, which is consistent with the conclusion in a previous study (15). Common recognized characteristics among women with BV and TV are the alteration of vaginal compositions, elevation of vaginal pH, and increase of bacterially produced metabolites such as sialidase, proteases, PLC and PLA_2_, etc., which degrade the mucus secreted from the cervix and facilitates the HPV virus adhering to and breaching the protective epithelial barrier (16). In addition, sialidase participates in the regulation of the innate immunity of the host and thus increases the susceptibility of HPV (17). Nevertheless, the specific relationship between HPV16/18 and vaginal microecology remains unclear. Some researchers hold that the comparatively high viral load may matter, but further studies on its mechanism are required. In the vagina of HPV-positive women, the reproduction of lactobacillus is further inhibited, thus shifting the profile of microorganisms and ultimately leading to the imbalance of the vaginal micro-environment (18). Hence, we can reach the conclusion that there is a relationship between HPV infection and vaginal micro-environmental imbalance. With a knowledge that imbalanced vaginal environment favors HPV infection, we can understand the pathogenesis of this viral infection and seek alternative prophylaxis.

Emerging evidence emphasizes that the vaginal micro-environment varies in women with different precancerous diseases, and the evolution of CIN is correlated with the presence of BV, TV, and unstable vaginal PH. A recent systematic review and network meta-analysis reported that barely half of patients with CIN could be attributed to BV infection, whereas Candida albicans was not a causative agent of cervical lesions (19). The study by Mania-Pramanik et al. (20) indicated a significant association between vaginal PH value and cervical dysplasia, and women with vaginal PH>4.5 were predisposed to cervical diseases. In our study, the severity of histological lesions was strongly relevant to abnormal PH value and vaginal cleanness. Lactobacillus inhibit colonization of BV-related bacterial species through maintenance of the acidic environment and production of corresponding bacteriocins (15). Hence, imbalanced PH value can induce growth of BV-associated taxa and potential pathogens such as Chlamydia trachomatis, Chlamydia trachomatis and Gardnerella vaginitis, leading to the inflammatory syndrome which is well-documented to promote the development of HPV infection and cervical neoplasia. Besides, abnormal vagina cleanness greatly increase the probability of BV and aerobic vaginitis (AV), finally participating in the pathogenesis of HPV infection (21). The above-mentioned indicators serve as both meaningful clues to vaginitis diagnosis and latent predictors for cervical lesions, and also provide reference for the development of adjuvant therapy with probiotics.

The present study is endowed with a few limitations. First, the uniformity among participants adversely affects the popularity of the research. Second, vaginal microecology can be dynamically modulated, and imperfect acquaintance with some exogenous factors (i.e., contraception, sexual intercourse, hygiene practices, etc.) lead to incomprehensive interpretation of the data. In general, we concluded that the presence of clue cell and sialidase are risk factors for HPV infection, and abnormal PH value and vaginal cleanness may be associated with the severity of precancerous lesions. Thus, indicators of vaginal discharge can be useful microbiological predictors of HPV infection and cervical diseases in some women. Furthermore, detection of vaginal secretion may be able to help the development of targets for micro-environmental modulation with probiotics. Well-powered dense-sampling longitudinal cohorts studies are required in the further to assess the implication of regulated vaginal microecology on HPV infection, cervical lesions and even disease recurrence, thus promoting risk stratification and helping with clinical decision making.

## Data availability statement

The raw data supporting the conclusions of this article will be made available by the authors, without undue reservation.

## Ethics statement

The studies involving humans were approved by Ethics Committee of the First Affiliated Hospital of Wenzhou Medical University. The studies were conducted in accordance with the local legislation and institutional requirements. The participants provided their written informed consent to participate in this study.

## Author contributions

LZ: Writing – review & editing. RF: Writing – original draft. YTH: Supervision, Writing – review & editing. JH: Data curation, Writing – review & editing. YH: Methodology, Writing – review & editing.
