# Supplementary figures and images for: Relationship between vaginal microecology and human papillomavirus infection as well as cervical intraepithelial neoplasia in 2,147 women from Wenzhou, the southeast of China
Source: Front Oncol. 2024 Jan 3;13:1306376. doi: 10.3389/fonc.2023.1306376 (PMC10791863; doi:10.3389/fonc.2023.1306376)

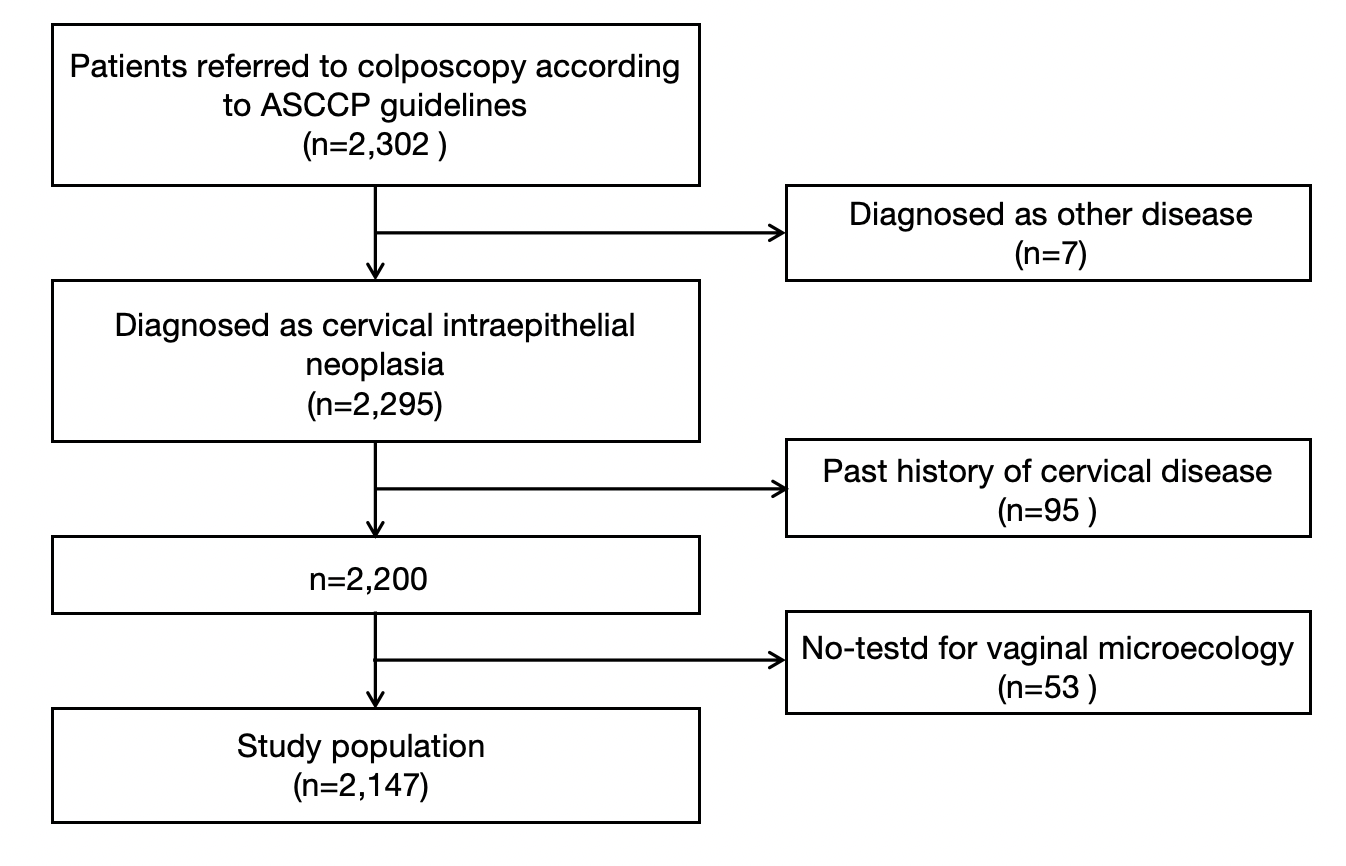

Supplement: Supplementary file 1 [file Image_1.tiff]
